# P-302. Changes in Antimicrobial Susceptibilities of Enterobacterales Causing Sepsis in Neutropenic Pediatric Patients with Underlying Hemato-oncologic Diseases

**DOI:** 10.1093/ofid/ofae631.505

**Published:** 2025-01-29

**Authors:** Yoon Kyung Cho, Ye Ji Kim, Jae Won Yoo, Hyun Mi Kang, Jae Wook Lee, Nack-Gyun Chung, Bin Cho, Dae Chul Jeong

**Affiliations:** The Catholic University of Korea, Seoul St. Mary's Hospital, Seoul, Seoul-t'ukpyolsi, Republic of Korea; The Catholic University of Korea, Seoul, Seoul-t'ukpyolsi, Republic of Korea; Seoul St. Mary's Hospital, Seoul, Seoul-t'ukpyolsi, Republic of Korea; The Catholic University of Korea, Seoul St. Mary's Hospital, Seoul, Seoul-t'ukpyolsi, Republic of Korea; The Catholic University of Korea, Seoul St. Mary's Hospital, Seoul, Seoul-t'ukpyolsi, Republic of Korea; The Catholic University of Korea, Seoul St. Mary's Hospital, Seoul, Seoul-t'ukpyolsi, Republic of Korea; The Catholic University of Korea, Seoul St. Mary's Hospital, Seoul, Seoul-t'ukpyolsi, Republic of Korea; The Catholic University of Korea, Seoul, Seoul-t'ukpyolsi, Republic of Korea

## Abstract

**Background:**

As neutropenia episodes in pediatric hemato-oncology often lead to sepsis and need antibiotics, monitoring resistance is crucial for empirical antibiotics choice. We analyzed antimicrobial susceptibility changes in Enterobacterales from sepsis pediatric patients at Seoul St. Mary’s bone marrow (BMT) center. Data from 2016-2020 and 2021-2023 were compared after shifting initial empirical antibiotics from piperacillin-tazobactam (PIP-TZ) to cefepime (CEF).

**Methods:**

This was a retrospective cohort study of sepsis patients at the pediatric BMT center of Seoul St. Mary’s hospital. Data from two time periods, 2016-2020 and 2021-2023, were compared, as the initial empirical antibiotic was switched from piperacillin-tazobactam (PIP-TZ) to cefepime (CEF).

**Results:**

Of 370 bacteremia cases, 70% (n=259) occurred during 2016-2020, and 30% (n=111) during 2021-2023. The proportion of sepsis by Enterobacterales increased, with 32.8% (n=85/259) during 2016-2020 and 57.6% (n=64/111) during 2021-2023 (*P* < 0.001). After antibiotic switch, the proportion of extended-spectrum beta-lactamases-producing *E.coli* and *K.pneumoniae* increased from 14.1% (n=10/71) to 30.9% (n=17/55) (*P* = 0.02). The overall susceptibilities of to third and fourth generation cephalosporins decreased: cefotaxime (78.3% to 49.3%, *P* < 0.001); ceftazidime (87.4% to 64.4%, *P* = 0.0046), and CEF (87.4% to 68.5%, *P* = 0.002). The overall susceptibilities to PIP-TZ increased: 78.2% to 83.6% (*P* = 0.30). For *P.aeruginosa*, the susceptibilities to PIP-TZ and CEF decreased: 78.6% to 66.7%, *P* = 0.53 and 85.7% to 77.8%, *P* = 0.62, respectively. Carbapenem-resistance was observed in *P.aeruginosa, A.baumannii* and *K.pneumoniae* during 2016-2020 while it was observed in *S.pneumoniae, K.pneumoniae, P.aeruginosa, E.cloacae* and *M.morganii* during 2021-2023. 3.5% (n=4/115) of gram negative sepsis patients had carbapenem-resistance in 2016-2020 while 10.9% (n=8/73) in 2021-2023 (*P* = 0.04).

**Conclusion:**

Cephalosporin resistance increased after the change of initial empirical antibiotics, while *P.aeruginosa* showed the opposite resistance. Continuous monitoring for any increase in carbapenem resistance is necessary.

**Disclosures:**

**All Authors**: No reported disclosures

